# Preliminary study of short-term outcomes and learning curves of robotic-assisted THA: comparison between closed platform robotic system and open platform robotic system

**DOI:** 10.1186/s12891-023-06895-9

**Published:** 2023-09-26

**Authors:** Teng-Feng Zhuang, Chong-Jie Wu, Si-Min Luo, Wen-Rui Wu, Jun-Yuan Chen, Zhen-Gang Zha, Song-Wei Huan, Ning Liu

**Affiliations:** 1 The First Clinical College, Jinan University, Guangzhou, 510632 China; 2 Department of Orthopedics, The First Affiliated Hospital, Jinan University, Guangzhou, 510632 China

**Keywords:** Total hip arthroplasty, Robotic-assisted surgery, Cup positioning, Learning curve

## Abstract

**Background:**

Both closed platform and open platform robotic-assisted total hip arthroplasty (THA) have recently been recommended as a viable treatment option for achieving accurate positioning of components. Yet, limited studies paid attention to the differences between the closed platform robotic system and the open platform robotic system. Hence, this study aimed to investigate clinical outcomes, radiographic outcomes, complication rates and learning curve of two systems.

**Materials and methods:**

We retrospectively included 62 patients (31 closed robotic system and 31 open robotic system) who underwent THA between February 2021 and January 2023. The demographics, operating time, cup positioning, complications and hip Harris score were evaluated. Learning curves of operation time was conducted using cumulative sum (CUSUM) analysis.

**Results:**

There were no differences in surgical time (76.7 ± 12.1 min vs. 72.3 ± 14.8 min), estimated blood loss (223.2 ± 13.2 ml vs. 216.9 ± 17 ml) and Harris Hip score (HHS) between closed platform robotic system and the open platform robotic system. The closed robotic system and the open robotic system were associated with a learning curve of 9 cases and 7 cases for surgical time respectively, based on the satisfying rate of Lewinnek’s safe zone outliers (1/31, 96.8%) and no occurrence of complication. Both robotic systems had significant reduction in overall surgical time, the duration of acetabulum registration, and estimated blood loss between learning phase and proficiency phase.

**Conclusion:**

The authors suggest that the surgical outcomes and safe zone outlier rate of the open robotic-assisted THA were similar to those of the closed robotic-assisted THA. These two robotic-assisted are associated with comparable learning curves and both have the precise positioning of acetabular component. From learning phase to proficiency phase, the rate of positions within the safe zone differed only marginally (88.9–100% vs. 85.7–100%) based on a rather low number of patients. This is not a statistically significant difference. Therefore, we suggest that THA undergoing with the robotic-assisted system is the relatively useful way to achieve planned acetabular cup position so far.

## Introduction

Total hip arthroplasty (THA) has been known as a successful treatment for end-stage hip disorders and has been related to high patient satisfaction [1]. Nevertheless, instability is a major complication following THA and remains the most common reason for revision THA in the United States [2]. Instability after THA leads to ascending medical costs by up to three hundreds percentage of the cost of a primary THA [3].

Regarding this issue, component positioning was considered as one of the critical factors by surgeons. The acetabular cup malposition is related to a series of inferior outcomes, including high incidence of dislocation, recurrent implant impingement, and accelerating liner wear [4]. In the past several decades, the robotic-assisted technology has been developed to enhance the surgical planning, optimize the component sizing, and improve the accuracy of component implantation [5].

Most authors reported that the robotic-assisted THA can accurately implant acetabular component within “safe zone” described by Lewinnek [6, 7]. Due to growing attention, currently, the market offers various designs and choices in terms of different concepts [8]. The robotic-assisted system can be classified as closed platform and open platform. The robotic-assisted system that matches with specific implants produced by the surgical robot provider is known as “closed platform”. In addition, the other one that matches with more various designs of implants produced by broader provider (not only the surgical robot provider) is known as “open platform”. According to the outcomes of the previous studies, both of them resulted in good component alignment and satisfying clinical outcomes [9, 10]. Although the “open platform” can use various designs of implant from different companies based on the patients’ anatomical features, it is partially lack of specificity and functionality [5]. The debate regarding these two types of the robotic system is still going on.

However, to the best of our knowledge, there are no studies directly comparing the outcomes of THA with the closed or open platform robotic system. In the present preliminary study, we aim to compare the learning curve, radiological and clinical outcomes between the closed robotic-assisted system and the open robotic-assisted system.

## Method

### Study design

This retrospective comparative analysis received approval from the Institutional Review Board of the Jinan University First Affiliated Hospital (No.KY-2023-016). Between February 2021 and January 2023, the data from 62 patients who performed robotic-assisted total hip arthroplasty (THA) were reviewed, including 31 consecutive patients who performed THA using the closed-platform MAKO system (Version 1.0, Stryker, Kalamazoo, MI, US) and 31 consecutive patients who performed THA using the open-platform Arthrobot system (Version 1.0, Montagne, Beijing, China). The Arthrobot is a novel haptic semi-active robotic-assisted arm which is compatible with various designs of implants from different companies. All of the surgeries were performed by single qualified senior surgeon (Ning Liu). The senior surgeon had more than 17 years of experience performing total hip arthroplasty and had collectively completed over 4000 total hip arthroplasties as of 2023. Furthermore, the senior surgeon completed the standard training program of both systems successfully. The standard training programs of both systems were comparable, including practicing one robotic-assisted THA in a Sawbone model and performing six robotic-assisted THA in patients under the supervision of the certificated surgeon. All included patients were informed and agreed to undergo the robotic-assisted THA. The patients who underwent simultaneous bilateral or revision THA were excluded. The patients who had history of prior surgery to the affected hip were excluded as well.

### Preoperative preparation

For all of the patients, preoperative computed tomography (CT) scans of the affected hip were acquired and preoperative planning was conducted by the Mako or the Arthrobot software. In both the Mako and the Arthrobot system, targeted acetabular cup positioning was 40 degrees for inclination 20 degrees for anteversion with adjusting variable pelvic tilt. The senior surgeon reviewed the plan before operation and selectively adjusted the component positioning relative to individual anatomical features.

### Surgical techniques

The posterolateral approach was used in all patients. After exposing into the hip joint, the surgeon performed dislocation of the joint and the osteotomy of femoral neck. The same procedure in both the MAKO and the Arthrobot system is as follow: Firstly, three pins were drilled into the anterior superior iliac spine in order to attach the pelvic array. Capturing posterior, anterior and superior landmarks in the rim of acetabulum with a probe electrode under optical navigation. After that, the acetabulum registration was proceeded to recognized 32 registration points and 8 verification points. In the registration stage, the goal is to establish the connection between the preoperative digital CT scan data and the real bone surface. The specific model of acetabulum was established based on the preoperative CT data aligned to the real bone when the precision of registration meets the criterion (error less than 1 mm). The acetabular reaming was guided by the robotic-assisted arm at target position. When the acetabulum preparation was finished, the surgeon implanted the acetabular component with the assistance of the robotic arm. The position of acetabular cup was verified by the robotic system. And the femoral stem was implanted manually and the stability of artificial hip joint was confirmed following reduction.

### Clinical and radiographic outcome assessments

We reviewed the surgical database and recorded the data related to operation, including general characteristics, surgical time, estimated blood loss, and complications. Harris Hip Score (HHS) was used to evaluate the clinical outcomes both preoperatively and in 24 h after the surgery [11]. The surgical time related to robotic-assisted systems was divided into four procedures, including: (1) pelvic array assembly; (2) registration of acetabulum; (3) acetabulum preparation; (4) implantation of acetabular component. Radiographic outcomes were assessed by postoperative anteroposterior radiograph and CT scan of the hip joint. Two observers independently measured the inclination and the anteversion of the acetabular component using postoperative CT data through the RadiAnt DICOM 2.2.9 Viewer (Medixant, Poznan, Poland). For measuring cup inclination and anteversion, the individual’s pelvic tilt was taken into account. The anterior superior iliac spines (ASIS) and the pubic tubercles were located based on the three-dimensional reconstructed CT images. A plane through them called anterior pelvic plane (APP) was employed for the pelvic coordinate system and adjustment of individual pelvic tilt. Due to the sagittal angle of APP is not always flat in a neutral (zero) position of the hip, we axially rotated until the bilateral ASISs in the same horizontal plane, and then the inter-teardrop line is considered as the mediolateral axis for measuring the cup alignment. When acetabular cup was implanted into the Lewinnek safe zone (45 degrees ± 10 degrees of inclination and 15 degrees ± 10 degrees of anteversion) [12], the procedure was considered as a success. In contrast, we considered it as an outlier of cup position.

### The learning curve

In the present study, we used the cumulative summation analysis (CUSUM) to evaluate the learning curve of robotic-assisted THA. The CUSUM provides the visualization of learning trends in the investigated technique of the surgeon and is a running total of the deviations from prespecified value [13]. The results were displaced in a graph with sequence of operation on the x-axis and the relative CUSUM value on the y-axis. A turning point in the curve is recognized as the symbol from a learning phase to a proficiency phase. The target surgical time was set by the average surgical time of THA guiding with the MAKO system and the Arthrobot system respectively. The CUSUM value would add the differences when the surgical time was longer than the average value, and the CUSUM value would reduce the differences when the surgical time was shorter than the average value.

### Statistical analysis

The numbers of cases (n) and frequencies (percentages) were calculated for categorical variables, and the means ± standard deviations (SDs) or medians were calculated for continuous variables. Student’s t-test and chi-square test were used to analyze the differences between two groups in continuous variables and categorical variables, respectively. We defined a P value < 0.05 as statistically significant. All statistical analyses were performed using SPSS software system (version 20.0, SPSS, Inc., Chicago, IL).

## Results

### Demographic

A total of 62 patients were enrolled, 31 patients underwent the closed-platform robotic-assisted THA, and the other 31 patients underwent the open-platform robotic-assisted THA. Among them, 24 patients were diagnosed as hip osteoarthritis and 7 patients were diagnosed as femoral neck fracture in the closed robotic system group, 22 patients were diagnosed as hip osteoarthritis and 9 patients were diagnosed as femoral neck fracture in the open robotic system group. No obvious differences in the demographics were detected between the closed robotic system group and the open robotic system group (Table 1).

**Table 1:** Patient Demographics

	Closed robotic system	Open robotic system	*P*
Number of patients	31	31	
Age	62.4 ± 8.6	64.7 ± 8.2	0.23
Gender(female/male)	17/14	18/13	0.79
BMI	21.2 ± 2.9	22.3 ± 2.7	0.16
Laterality(left/right)	16/15	17/14	0.79
Diagnosis			0.56
Hip osteoarthritis	24	22	
Femoral neck fracture	7	9	

*P < 0.05

### Clinical and radiographic outcomes

**Fig. 1:**
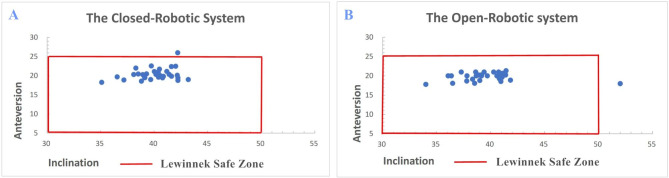
The scatterplot of the acetabular component positioning in the closed robotic-assisted THA (**A**) and the open robotic-assisted THA (**B**)

Mean surgical times were comparable in two groups with no significant differences (76.7 ± 12.1 min vs. 72.3 ± 14.8 min). Likewise, no significant differences about estimated blood loss between two groups (223.2 ± 13.2 ml vs. 216.9 ± 17 ml). Both robotic system implanted component accurately in Lewinnek safe zone (96.8% vs. 96.8%) (Fig. 1). Mean HHS was significantly improved after surgery compared to mean preoperative HHS in both groups (51.1 to 79.6 vs. 46.5 to 81.4) (Table 2).

**Table 2:** Clinical and radiographic outcomes of the robotic-assisted THAs

Variables	Closed robotic system	Open robotic system	*P*
Mean surgical time, min	76.7 ± 12.1	72.3 ± 14.8	0.29
Mean estimated blood loss, ml	223.2 ± 13.2	216.9 ± 17	0.11
Positioning of cup			
Mean cup inclination, °	40.1 ± 1.84	39.2 ± 1.97	0.07
Mean cup anteversion, °	20.4 ± 1.6	19.8 ± 0.97	0.06
Lewinnek’s safe zone, %	96.8%	96.8%	
HHS			
Mean preoperative HHS	51.1 ± 10.5	46.5 ± 13.1	0.13
Mean postoperative HHS	79.6 ± 11.9	81.4 ± 12.0	0.56
Mean change in HHS	28.5 ± 16.7	34.9 ± 17	0.14
Complications	0	0	

*P < 0.05

### Learning curve

**Fig. 2:**
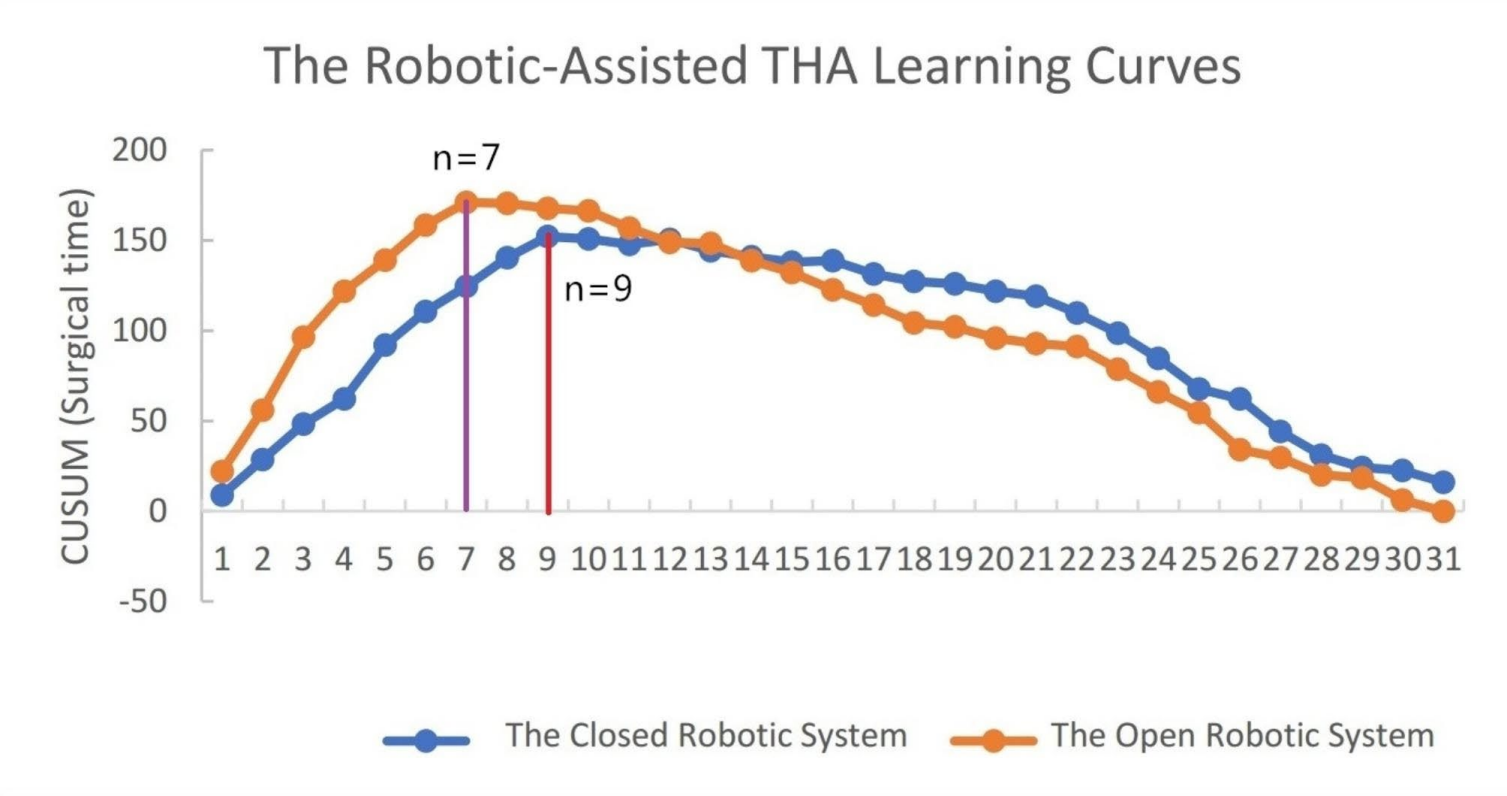
The learning curve based on CUSUM analysis of surgical time in the closed robotic-assisted THA and the open robotic-assisted THA

Regarding the CUSUM analysis of the surgical time, the inflexion point was detected in the 9th case in the closed robotic system. Additionally, the inflexion point of the learning curve of the open robotic system was detected in the 7th case (Fig. 2). These curves signaled competency after 9 surgeries for the closed robotic system and 7 surgeries for the open robotic system.

### Learning phase versus proficiency phase

We compared the differences in mean surgical time, surgical time related to robotics, mean estimated blood loss, positioning of cup, and complications between the learning phase and the proficiency phase (Table 3). In both closed robotic system and open robotic system, the overall surgical time in the proficiency phase were significantly shorter than that in the learning phase (69.78 ± 5.61 min vs. 91.3 ± 8.12 min, P < 0.001). Nevertheless, not all durations of procedures related to robotic assisted system have been decreased significantly between two phases. As experience of robotic THA accumulates, the durations of registration of acetabulum and acetabulum preparation had an obvious reduction in closed robotic system (12.35 ± 2.24 min vs. 8.62 ± 2.95 min, and 10.11 ± 2.85 min vs. 7.78 ± 2.79 min). For open robotic system, only the registration of acetabulum required less time in the proficiency phase compared to the learning phase (12 ± 2.18 min vs. 8.93 ± 2.33 min). The proportion of cup positioning within Lewinnek’s safe zone were changed from 88.9 to 100% and 85.7–100% between two phases in closed robotic system and open robotic system respectively.

**Table 3:** Comparison of the clinical and radiographic outcomes of the robotic-assisted THAs

Variables	Learning phase (Closed system)	Proficiency phase (Closed system)	*P*	Learning phase (Open system)	Proficiency phase (Open system)	*P*
Mean surgical time, min	91.3 ± 8.12	69.78 ± 5.61	< 0.001*	93.88 ± 12.57	65.16 ± 5.88	< 0.001*
Surgical time related to robotics						
Pelvic array assembly	8.11 ± 1.27	6.82 ± 2.02	0.09	8.44 ± 1.24	7.07 ± 1.95	0.06
Registration of acetabulum	12.35 ± 2.24	8.62 ± 2.95	< 0.05*	12 ± 2.18	8.93 ± 2.33	< 0.05*
Acetabulum preparation	10.11 ± 2.85	7.78 ± 2.79	< 0.05*	9.33 ± 3.12	7.21 ± 2.51	0.06
Implantation of component	7.33 ± 2.69	5.92 ± 1.97	0.11	6.89 ± 3.02	5.5 ± 2.63	0.21
Mean estimated blood loss, ml	236.05 ± 5.83	197.16 ± 11.12	< 0.001*	237.37 ± 13.72	209.75 ± 11.39	< 0.001*
Positioning of cup						
Mean cup inclination, °	40.74 ± 1	39.73 ± 2.08	0.16	38.82 ± 2.04	39.28 ± 1.98	0.58
Mean cup anteversion, °	20.71 ± 2.04	20.24 ± 1.28	0.44	19.45 ± 1.07	19.86 ± 0.94	0.31
Lewinnek’s safe zone, %	88.9%	100%		85.7%	100%	
Complications	0	0		0	0	

*P < 0.05

## Discussions

The robotic-assisted THA has been applied clinically for several years, claiming to enhance preoperative planning, improve the accuracy of the component positioning and restore the biomechanical features of the hip joint [9, 14]. A growing body of clinical studies supported that the robotic-assisted THA conducted accurate alignment and superior functional outcomes comparing with manual THA [1, 15, 16]. Different robotic-assisted systems have unique features used to make preoperative plan and conduct the surgery. The Mako robotic-assisted arm is a widely applied closed-platform robotic system worldwide [17]. The closed robotic-assisted system allows limited selection of implants which is not able to utilize different designs of implant according to patients’ characteristics [5]. To overcome this limitation, some robotic-assisted systems provide relatively large scale of freedom of selecting implants that are known as the open platform robotic system. However, there were some problems in the early generation open robotic systems. The CASPAR was considered as to have a lack of specificity and predictive value because it is imageless system and lacking the ability to adjust base on individual anatomical variations [18]. The ROBODOC was related to the disadvantages about the surgical approach and the trauma of soft tissue structures [19].Recently, a novel image-based open robotic-assisted system named Arthrobot is available in clinical practice. There currently is no consensus whether the image-based open robotic-assisted system has the same degree of accuracy compared with the image-based closed system. Therefore, we conducted the present study to determine whether there are any differences in short-term outcome between THA performed using the closed robotic-assisted system versus the open robotic-assisted system.

The most interesting finding of the present study was that the senior surgeon demonstrated competence in the closed robotic-assisted THA after an experience of 9 procedures and in the open robotic-assisted THA after an experience of 7 procedures, based on the satisfying rate of Lewinnek’s safe zone outliers (1/31, 96.8%) and no occurrence of complication. Kayani et al. [20] demonstrated that the closed robotic-assisted system was associated with learning curve of 12 cases in terms of operative time. The study by Sugano [21] suggested that surgeon training was an important issue for open imageless robotic-assisted system due to its steep learning curve. Conversely, in our study, the learning curve for open robotic-assisted system was slightly shorter than for the close robotic-assisted system. It should be mentioned that two types of robotic-assisted THA were conducted by the same surgeon. The previous experiences of the close robotic-assisted THA could help to accelerate the learning curve of the later open robotic-assisted THA. On the other hand, the open robotic-assisted system we used in this study is image-based, instead of the imageless system as it was used in the previous study.

In the present study, both robotic-assisted systems implanted acetabular component accurately within Lewinnek’s safe zone. For getting precise cup position, undergoing THA with robotic arm could be an available way. Kayani et al. [20] demonstrated that the proportion of acetabular prosthesis in Lewinnek’s safe zone was higher in robotic-assisted THA group (98%) than in the conventional THA group (68%). The reasons for accurate implantation of the acetabular component are likely multifactorial. Guo et al. [10] found that the postoperative measurement was approximately consistent with preoperative planning in the robotic-assisted system. They stressed the crucial role of detailed preoperative planning in obtaining ideal cup position. In another report on the accuracy of component installment in robotic-assisted THA, the differences between intraoperative data on component position and radiographic data on component position were not significant. This indicates that surgeons should remain vigilant to utilize intraoperative data to predict the actual component position [22]. Moreover, accurate reaming by using single-size reamer and precise component installation with robotic-arm may have contributed partially to the obviously low deviation in component alignment from the target position in the robotic-assisted THA [23].

The robotic-assisted THA seemingly requires additional surgical time, which can be one of the barriers for the use of this method [24]. However, a systematic review conducted a pooled analysis and reported that there is no significant difference in surgical time between the robotic-assisted THA and manual THA [25]. In the present study, there was a significant reduction of surgical time after passing the learning phase. The gradual acquirement of acetabulum registration and acetabulum preparation skills contributed to the significant reduction in overall time. The most time-consuming procedure was the acetabulum registration which occupied 10–15% of surgical time. After passing the learning curve, the consuming time in the acetabulum registration decreased by 30%. There are several skills that help to improve the accuracy of acetabulum registration: (1) distributing three landmarks as an isosceles triangle in anterior, posterior, and superior rim of acetabulum; (2) adjusting the retractor when registering the points at the anterior rim of acetabulum in order to expose sufficiently; (3) ensuring that the probe is contacting the bone instead of cartilage or soft tissue [26]. Therefore, the additional consuming time of robotic-assisted system may be offset by the increasing proficiency of the procedures as much as possible. As results related to robotic-assisted system become understood better, we believe the benefits of robotic-assisted system outweigh its extra cost of surgical time.

The present study had several limitations. The major limitation of present study was the relatively small sample size, it was also related to the limited patient population, but the two groups were relatively comparable for general characteristics. Therefore, the power of the data analysis may not be entirely sufficient to arrive at a conclusion. Yet, there are no studies compare the differences between open robotic-assisted THA and closed robotic-assisted THA. We suggest that the results of present study offer valuable evidence. In addition, the present study was limited in that the study design was retrospective and nonrandomized instead of prospective and randomized. In future, more prospective and randomized studies regrading this issue are necessary to provide more information. Thirdly, the present study addressed the comparison between one specific type of an open platform system with one specific type of closed platform system. The results may not be entirely applicable for all types of open platform system or closed platform system. Fourthly, there are no separate assessments of cup orientation for the robotic-assisted THA. The application of Lewinnek safe zone may lead to potential inconsistency. Finally, the single surgeon has a high-volume THA practice in present study. The learning curve may not be entirely applicable for all surgeons.

## Conclusion

In conclusion, this study demonstrated that the surgical outcomes and safe zone outlier rate of open robotic-assisted THA were comparable to those of closed robotic-assisted THA. Moreover, the learning curve of THA by the open robotic-assisted system was similar to THA by the closed robotic-assisted system although the open robotic-assisted system used more various designs of component. We believe that THA undergoing with the robotic-assisted system is one of the most reliable methods to achieve accurate component position up to date, even in learning phase.

## Data Availability

All data included in this study are available upon request by contact with the corresponding author.
